# S100A alarmins and thymic stromal lymphopoietin (TSLP) regulation in severe asthma following bronchial thermoplasty

**DOI:** 10.1186/s12931-023-02604-1

**Published:** 2023-11-23

**Authors:** Pierre-Alexandre Gagnon, Martin Klein, John De Vos, Sabrina Biardel, Andréanne Côté, Krystelle Godbout, Michel Laviolette, Catherine Laprise, Said Assou, Jamila Chakir

**Affiliations:** 1 Centre de Recherche, Institut Universitaire de Cardiologie et de Pneumologie de Québec-Université Laval (IUCPQ-UL), 2725 Chemin Sainte-Foy, Québec, QC G1V 4G5 Canada; 2 IRMB, Univ Montpellier, INSERM, CHU Montpellier, Montpellier, France; 3 Département des Sciences Fondamentales, Université du Québec à Chicoutimi (UQAC), Saguenay, QC Canada

**Keywords:** Alarmin, Bronchial thermoplasty, Severe asthma

## Abstract

**Rationale:**

Severe asthma affects a small proportion of asthmatics but represents a significant healthcare challenge. Bronchial thermoplasty (BT) is an interventional treatment approach preconized for uncontrolled severe asthma after considering biologics therapy. It was showed that BT long-lastingly improves asthma control. These improvements seem to be related to the ability of BT to reduce airway smooth muscle remodeling, reduce the number of nerve fibers and to modulate bronchial epithelium integrity and behavior. Current evidence suggest that BT downregulates epithelial mucins expression, cytokine production and metabolic profile. Despite these observations, biological mechanisms explaining asthma control improvement post-BT are still not well understood.

**Objectives:**

To assess whether BT affects gene signatures in bronchial epithelial cells (BECs).

**Methods:**

In this study we evaluated the transcriptome of cultured bronchial epithelial cells (BECs) of severe asthmatics obtained pre- and post-BT treatment using microarrays. We further validated gene and protein expressions in BECs and in bronchial biopsies with immunohistochemistry pre- and post-BT treatment.

**Measurements and main results:**

Transcriptomics analysis revealed that a large portion of differentially expressed genes (DEG) was involved in anti-viral response, anti-microbial response and pathogen induced cytokine storm signaling pathway. S100A gene family stood out as five members of this family where consistently downregulated post-BT. Further validation revealed that S100A7, S100A8, S100A9 and their receptor (RAGE, TLR4, CD36) expressions were highly enriched in severe asthmatic BECs. Further, these S100A family members were downregulated at the gene and protein levels in BECs and in bronchial biopsies of severe asthmatics post-BT. TLR4 and CD36 protein expression were also reduced in BECs post-BT. Thymic stromal lymphopoietin (*TSLP*) and human β-defensin 2* (hBD2)* were significantly decreased while no significant change was observed in *IL-25* and *IL-33*.

**Conclusions:**

These data suggest that BT might improve asthma control by downregulating epithelial derived S100A family expression and related downstream signaling pathways.

**Supplementary Information:**

The online version contains supplementary material available at 10.1186/s12931-023-02604-1.

## Introduction

Asthma is a chronic inflammatory airway disease with increasing prevalence affecting over 300 million people worldwide. Severe asthma only affects approximately 5 to 10% of asthmatic patients. However, this proportion of asthmatics represents a significant healthcare challenge and contributes to up to half of direct asthma related costs. They respond poorly to standard asthma treatments and are more exposed to oral corticosteroids to achieve acceptable control with significant adverse outcomes associated with deterioration in quality of life [1].

According to current clinical standards, biologic treatment will be considered in such patients (GINA). Nevertheless, most of currently available biologics target T2 inflammation and are more effective in patients harboring features of such inflammation [2]. Even though, some patients might not be eligible or might not benefit from biologics therapy. For instance, a study evaluating response to mepolizumab (anti-IL5) of patients with high blood eosinophils reported cessation of treatment in about 14% of patients mainly because of failure to improve asthma control or based on clinician’s decision [3].

An alternative to biologics therapy lays in bronchial thermoplasty (BT) which has been preconized in severe asthma patients unresponsive or ineligible to biologics, and therefore has been utilized in patients harboring features of T2-high and T2-low inflammation [2, 4, 5]. This approach consists in a single delivery of radiofrequency thermal energy in airways of 3–10 mm in diameter. Clinical trials showed that BT is safe and effective to reduce asthma exacerbations, emergency department visits and hospital admissions and improves quality of life for up to 10 years in patients with severe asthma treated with BT [6–10]. Observational and meta-analysis studies have further showed that BT induces clinical improvements comparable to biologics [11–13].

BT is the only treatment approach known to modulate airway remodeling, a characteristic asthma-related feature. BT induces airway smooth muscle (ASM) ablation occurring the first weeks after treatment and persisting for over a decade [14–20]. Subepithelial membrane thickening, and airway associated nerve fibers are similarly reduced [14–17, 20]. We and others have further showed that airway epithelium may be a target of BT. For instance, we showed that the structure of airway epithelium was improved following BT treatment and that airway epithelium regeneration in response to BT-induced epithelial injury decreased mucin expression, improved goblet cell metaplasia and regenerated the ciliated cell layer, providing a durable improvement of airway epithelium integrity [21, 22]. BT also seems to modulate epithelial metabolic profile leading to a shift from a glycolysis-biased gene expression profile to an oxidative phosphorylation-based profile [23]. BT further seems to modulate the inflammatory profile of the airway epithelium [17, 21, 24].

Despite these observations, biological mechanisms explaining asthma control improvement following BT are still not well understood. This along with recent success of Tezepelumab targeting the epithelial derived thymic stromal lymphopoietin (TSLP) to improve asthma control regardless of T2 inflammation reemphasizes the cardinal role of the airway epithelium in asthma pathophysiology [25]. In this study, we assess whether BT affects gene signatures relevant to severe asthma pathophysiology in bronchial epithelial cells (BECs). To do so, we evaluate the transcriptome of BECs obtained pre- and post-BT treatment. We further validate protein expression of differentially expressed genes in bronchial biopsies pre- and post-BT treatment.

## Materials and methods

### Subjects’ evaluation, BT procedure and biopsy processing

Twenty patients having suboptimal asthma control despite daily doses of at least 500 µg of fluticasone and at least 100 µg of salmeterol (or equivalents) were included in a clinical BT protocol at the Quebec Heart and Lung Institute asthma clinic. Bronchial biopsies were collected before BT and during a follow-up evaluation occurring at 12–48 months (mean 23 months) after treatment. Bronchial biopsies were also collected from non-smoking participants without asthma nor allergy. Further details about inclusion criteria, BT procedure and biopsy processing are provided elsewhere [13, 18]. The Ethics Committee at the Quebec Heart and Lung Institute approved the study. Participants were asked for consent to undergo bronchial biopsies and signed an informed consent form.

### BECs culture

BECs isolated from bronchial biopsies of healthy controls (n = 7) and severe asthmatics pre-BT (n = 12) and 12–18 months post-BT (n = 6) were cultivated as we previously reported [26]. A representative immunofluorescence staining of epithelial cells with pan-cytokeratin antibody is showed in Additional file 4: Fig. S1. Cells were cultured until they reached over 90% confluence and were then collected in the appropriate lysis buffer and stored at − 80 °C until needed. More details are provided in Additional file 1.

### Microarray analysis, data processing and bioinformatic analysis

Transcript levels data was generated using Clariom™S HT, human (Thermo Fisher Scientific, Massachusetts, USA). The expression pattern of the transcripts was analyzed using the robust multi-array average (RMA) algorithm and to construct a principal component analysis (PCA), the volcano plot and the heat-map. Different transcript levels with a significant *p*-value < 0.05 and fold changes of 2 or higher and of -2 or lower were retained. The gene ontology (GO) enrichment analysis, the pathways, and networks of differentially expressed transcripts between pre- and post-BT samples were analyzed using Ingenuity Pathway Analysis (IPA) software (IPA; Qiagen Inc., http://www.ingenuity.com). All our data are accessible at the gene expression Omnibus (GEO) repository (https://www.ncbi.nlm.nih.gov/geo) with the provisional accession series number GSE216617. More details are provided in Additional file 1.

### Real-time quantitative PCR analysis

One μg of total RNA was reverse transcribed and used for qRT-PCRs. Relative gene expression was normalized on GAPDH of healthy individuals’ expression using ΔΔCt method [27]. More details are provided in Additional file 1.

### Protein extraction and Western blot

Twenty to 50 μg of total proteins was loaded on 12.5% acrylamide gel for SDS-PAGE and transferred on nitrocellulose membranes. Protein expression was measured by densitometry using Image Lab (Biorad, California, USA) and normalized to β-actin expression. More details are provided in Additional file 1.

### Immunohistochemistry (IHC)

Five µm sections of paraffin embedded bronchial biopsies were stained using the EXPOSE mouse and rabbit specific HRP/AEC detection IHC kit (Abcam, Cambridge, United-Kingdom) according to manufacturer’s instructions. Positively stained epithelial area was measured as a percentage of total epithelial area using ImageJ (NIH, V1.53f51) as we previously reported [21]. More details are provided in Additional file 1.

### Statistical analysis

Statistical analysis for qRT-PCR, western blots and immunohistochemistry were conducted using GraphPad Prism Version 9.0 statistical software (GraphPad Software). Data are expressed as mean ± standard error. Comparisons between groups when data were normally or not normally distributed were made with the Student’s *t* test or the Mann–Whitney *U* test. For correlation analysis Pearson or Spearman tests were used. Significance was accepted at a value of *P* < 0.05.

## Results

### Subjects’ clinical characteristics before and post-BT

Clinical characteristics of subjects are described in Table 1. Post-BD FEV_1_ values did not change over time: 2.66 ± 0.18 L and 2.83 ± 0.19 L at baseline and at ≥ 12 months post-BT respectively. Four subjects were taking oral prednisone before BT and their doses decreased from a mean of 18.1 ± 5.5 to 6.3 ± 2.4 mg daily at long-term follow-up. The doses of inhaled corticosteroid (ICS) decreased from 1235 ± 175 µg before BT and 938 ± 166 µg of fluticasone or equivalent (p = 0.034) after-BT. The number of severe exacerbations decreased (p = 0.018) and the asthma control scoring system (ACSS) score, a local equivalent of ACQ [28], improved from 71 ± 4% to 88 ± 3% at long-term post-BT follow-up (p = 0.0004). Healthy controls were non-smokers, had no allergy or asthma or other inflammatory diseases. Their mean PC_20_ was 107 ± 29 mg/ml and their mean FEV_1_ was 3.50 ± 0.38 L; 93 ± 7 of % predicted.

**Table 1:** Subjects’ clinical characteristics

	Pre-BT	Post-BT	p-value
Number of subjects	20	20	
Months since BT*	–	23 (15)	
Age (years)^†^	47 ± 2	49 ± 2	
Sex (M/F)	8/12	8/12	
Smoking status (no/ex/yes)	13/6/1	13/6/1	
Atopy (no/yes)	6/14	6/14	
Inhaled corticosteroid dose (µg)^†,§^	1275 ± 175	938 ± 166	0.035
Median (range)	1375 (0–3000)	750 (0–3000)	
Long acting β_2_-agonist (µg)^†,ll^	114 ± 14	102 ± 13	0.45
Median (range)	100 (0–250)	100 (0–200)	
Oral corticosteroids (n)	4 (7.5–30.0)	3 (5.0–10.0)	0.25
Omalizumab (n)	3	3	> 0.99
Mepolizumab (n)	1	2	0.60
Montelukast (n)	9	8	> 0.99
Post-BD FEV_1_ (L)^†^	2.66 ± 0.18	2.83 ± 0.19	0.17
Count of severe exacerbations^‡^	1 (0–10)	0 (0–2)	0.019
ACSS^†^	71 ± 4	88 ± 3	0.0004

*Mean (median)

^†^Mean ± standard error

^‡^Median (range)

§Fluticasone propionate or equivalent

^ll^Salmeterol or equivalent

### Transcriptomics analysis

**Fig. 1:**
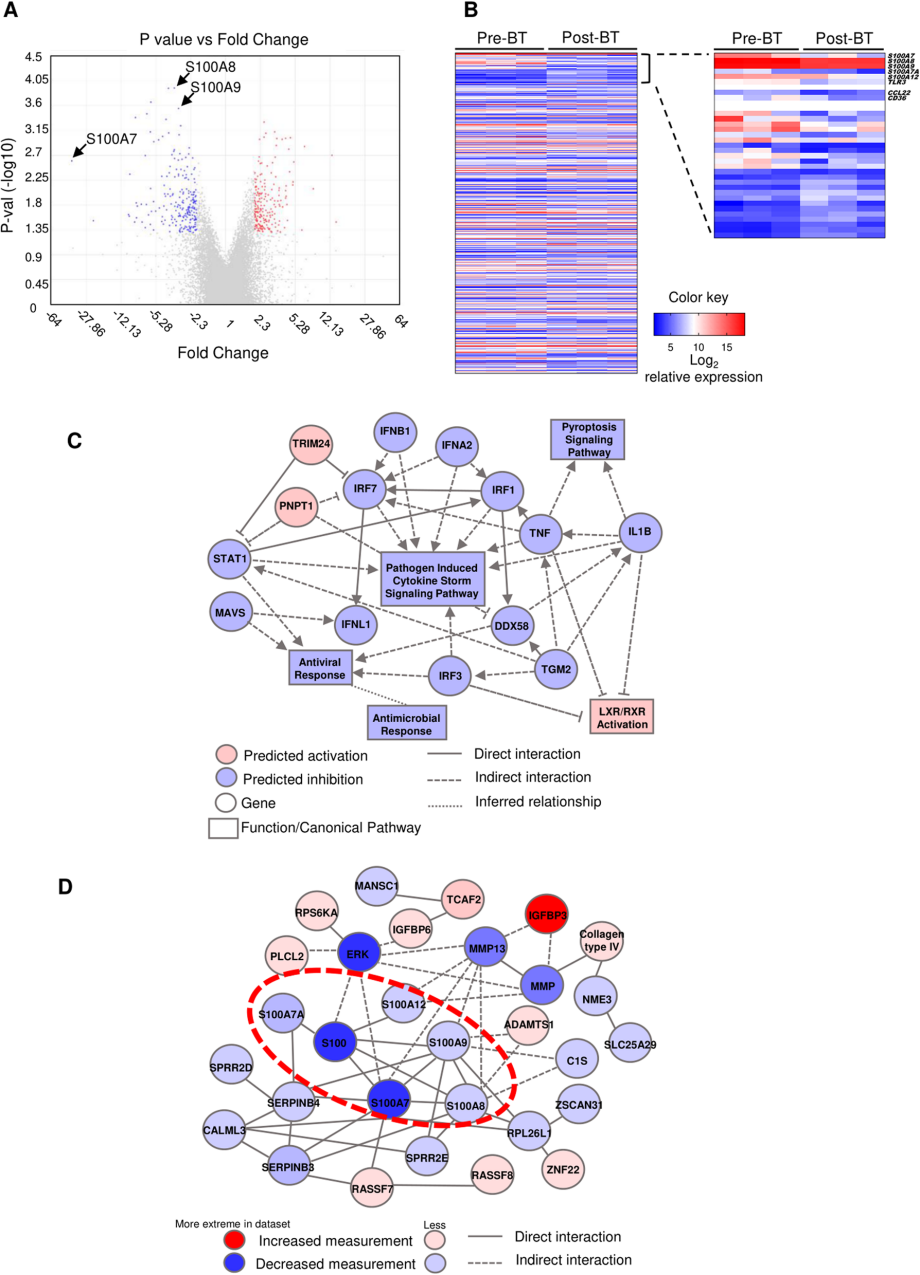
Transcriptomic analysis of BECs of severe asthmatics pre- and post-BT measured using microarray. **A** Volcano plot showing the distribution of transcript expression P values and fold changes. All transcripts were tested using the transcriptome analysis console (TAC) software. **B** Heat map. The molecular signatures pre-BT and post-BT were visualized by hierarchical clustering based on the differential transcripts’ expression. **C** Interaction network constructed using the ingenuity pathway analysis software. Nodes shaded in pink or red represent genes that are upregulated, and blue nodes are downregulated post-BT. A solid line represents a direct interaction and a dotted line an indirect interaction. **D** Several S100A family genes were interconnected in the same network in the post-BT signature. The color intensity indicates the degrees of up-regulation or down-regulation

We compared by microarrays the transcriptome profile of epithelial cells obtained from the same donors before and after BT. We identified 421 genes that were differentially expressed between pre- and post-BT with a twofold change cut-off and p-value < 0.05 (Fig. 1A). Two hundred twenty-two genes were downregulated and 199 upregulated post-BT (Additional file 2: Table S3). The dendrogram of hierarchical clustering analysis showed that cells before and post-BT treatment expressed specific molecular signatures (Fig. 1B). Mapping of the samples in a 3-dimensional space using principal component analysis (PCA) showed that BECs pre-BT were separated from BECs post-BT based on their gene expression profile (Additional file 5: Fig. S2A).

Heatmap plot based on the differentially expressed genes (DEG) separated the effects of BT treatment on cell samples and depicted overrepresentation of genes involved in relevant pathways among the genes differentially expressed pre- and post-BT. DEGs were then functionally categorized using the IPA software. A large portion of DEGs was involved in anti-viral response, anti-microbial response and pathogen induced cytokine storm signaling pathway (Fig. 1C). Further analysis revealed that the interleukin (IL)-6,8,12,13,17, toll-like receptor (TLR), interferon, p38 MAP kinase, wound healing, inducible nitric oxide synthase (iNOS), mammalian target of rapamycin (mTOR), production of nitric oxide and reactive oxygen species in macrophages, signal transducer and activator of transcription 3 (STAT3) and inflammasome signaling canonical pathways were modulated post-BT (Additional file 5: Fig. S2B and Additional file 3: Table S4). IPA software was used to build an interaction network. Strikingly, the interaction network showed that BECs pre- and post-BT signatures were highly enriched in genes associated to S100A alarmin family. These genes were downregulated in post-BT (Fig. 1A, B). Remarkably, numerous genes from S100A alarmin family such as *S100A7*, *S100A8*, *S100A9* were connected and formed a relevant network (Fig. 1D). Interestingly, these candidate genes are predicted downstream IL-17 signaling pathway and related to antimicrobial response (Additional file 5: Fig. S2C).

### S100A family gene and protein validation

**Fig. 2:**
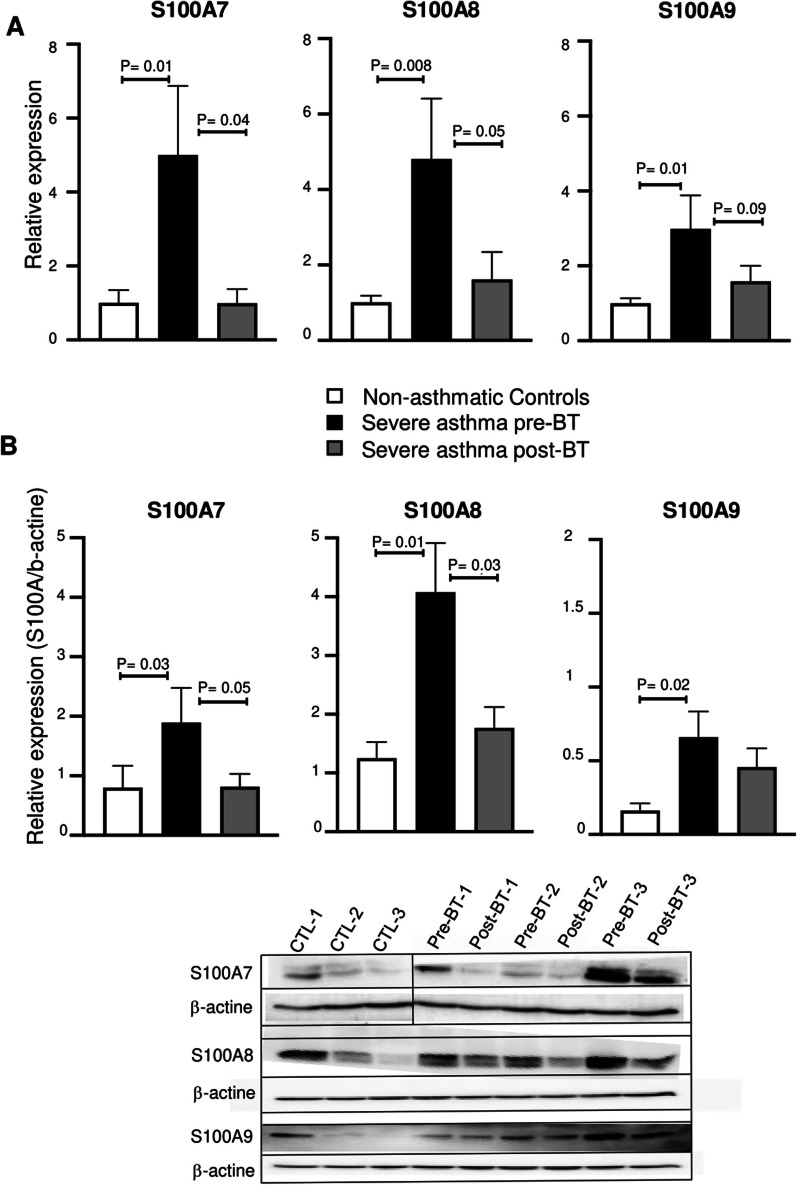
Validation of S100A family gene and protein expressions in BECs of non-asthmatic-controls and severe asthmatics pre- and post-BT. **A** Bar plots representing qPCR data. **B** Bar plot representing Western Blot data on top with representative blots below

To validate these microarray data, qRT-PCR was performed on an independent collection of BECs samples obtained from severe asthma patients pre- and post-BT treatment. We first documented baseline expression of *S100A7*, *S100A8* and *S100A9* genes in severe asthma and compared to BECs obtained from healthy controls. Figure 2A shows that *S100A7*, *S100A8* and *S100A9* genes were highly expressed in BECs of severe asthmatics in comparison to those of healthy controls. This expression is on average fivefold for *S100A7* (p = 0.01), 4.8-fold for *S100A8* (p = 0.008) and threefold for *S100A9* (p = 0.01) higher in BECs from severe asthmatics in comparison to healthy control. Post-BT data showed a significant decrease compared to pre-BT values in *S100A7 (*fourfold decrease, p = 0.03), and *S100A8 *(3.6-fold decrease, p = 0.05)*.* The decrease is less marked for *S100A9* gene expression (1.6-fold decrease, p = 0.09) (Fig. 2B).

At protein level, expression of all three S100 family proteins was highly upregulated in BECs of severe asthmatic patients in comparison to healthy controls (p = 0.03, p = 0.01, p = 0.02 respectively). BT treatment reduced protein levels of S100A7, S100A8 (p = 0.05, p = 0.03) and did not affect S100A9 in comparison to pre-BT values (Fig. 4B).

### S100A family epithelial in bronchial biopsies of severe asthmatics

**Fig. 3:**
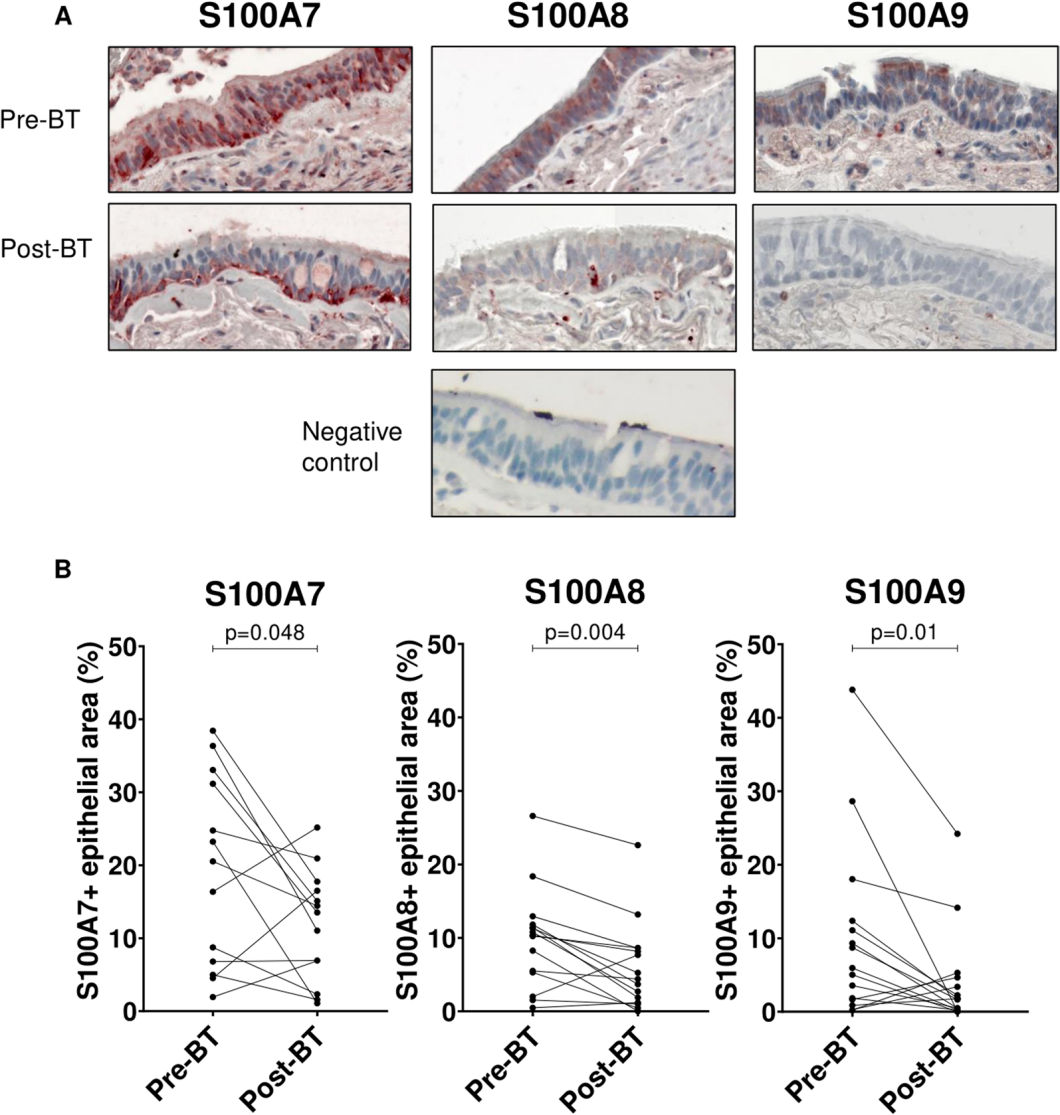
S100A family protein expressions in tissues of severe asthmatics pre- and post-BT.** A** Representative immunostainings for S100A family proteins.** B** Graphical representations of immunostaining quantification. S100A family protein expression is represented as % of epithelial area

S100A7, S100A8 and S100A9 expression in bronchial tissues were evaluated by immunostaining of bronchial biopsies obtained pre- and post-BT treatment. Figure 3A shows representative immunostaining for S100A7, S100A8 and S100A9 in bronchial biopsies with high expression pre-BT and reduced expression post-BT. Mean epithelial positive area of each S100A decreased post-BT: 19% to 11.8% for S100A7 (p-value = 0.048); 9.8% to 6% for S100A8 (p-value = 0.004) and 9.3% to 4% for S100A9 (p-value = 0.012) (Fig. 3B).

The decrease of S100A7 and S100A9 epithelial expressions following BT correlated with their pre-BT epithelial levels of expression (p-values = 0.0006 and < 0.0001, respectively). A similar tendency towards correlation was observed for S100A8 (p-value = 0.08 (Additional file 6: Fig. S3A). Epithelial expression of S100A7 and S100A8 pre-BT negatively correlated with ACCS score (r = − 0.6; p-value = 0.03 and r = − 0.7; p-value = 0.006 respectively) suggesting that higher expression of these proteins might indicate a poorer asthma control (Additional file 6: Fig. S3B).

### S100A family receptors and other alarmins

**Fig. 4:**
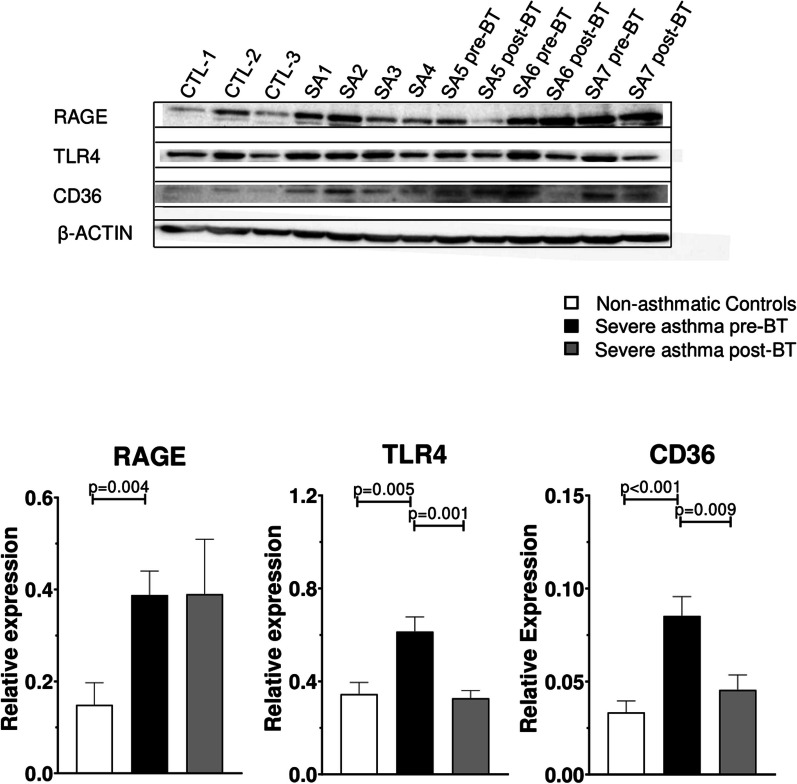
Expression of S100A family receptors in BECs. Western Blots of selected S100A family receptors (RAGE, TLR4, CD36) on top with associated graphical representations below

We found by Western Blot experiments that S100A family receptors were highly expressed in severe asthmatic BECs. The expression is on average of 2.6-fold for RAGE (p = 0.004), 1.8-fold for TLR4 (p = 0.005) and 2.6-fold for CD36 (p < 0.001). We further found a significant decrease in TLR4 and CD36 expression post-BT (p-values of 0.001 and 0.01 respectively) and no change in RAGE expression (Fig. 4).

We also evaluated by qRT-PCR the effect of BT on the expression of *IL-25*, *IL-33, TSLP* and *hBD2*; typical asthma-related alarmins or antimicrobial peptides in BECs. We observed a significant decrease in *TSLP* (p = 0.02) and *hBD2* (p = 0.02), while no significant change was observed in *IL-25* and *IL-33* (Additional file 7: Fig. S4).

## Discussion

This study showed that transcriptomic analysis of BECs identified a downregulation of inflammation with the modulation of asthma-related relevant canonical pathways such as IL-13 and S100A alarmins downstream of IL-17 signaling as potential gene family regulated by BT treatment. We found that BT downregulated *S100A7*, *S100A8* and *S100A9* gene expressions of cultured BECs. This downregulation was further validated at gene and protein levels in BECs and bronchial biopsies of the same patients. Moreover, this downregulation of S100A family 1-year or more post-BT paralleled the reduction of asthma exacerbations and better asthma control. We showed that S100A family receptors; RAGE, TLR4 and CD36, are upregulated in severe asthmatic BECs and that BT induces a downregulation of TLR4 and CD36 expression. Finally, gene expressions of *TSLP* and *hBD2*, are downregulated post-BT in BECs.

The S100A family proteins of interest here are immunomodulatory, antioxidant and calcium/zinc binding proteins mainly expressed by leucocytes of myeloid origin (neutrophils, monocytes) and/or epithelial cells (basal respiratory cells, keratinocytes) [29, 30]. Higher expressions of S100A8, S100A9 and/or calprotectin (S100A8/A9 complex) were reported in sputum, bronchoalveolar lavage (BAL) and serum of asthmatic patients when compared to healthy controls [31–37]. Decaesteker et al. reported that there was no difference in serum calprotectin levels between asthmatics patients with high sputum eosinophilia and patients with sputum neutrophilia. However, severe asthmatic patients seemed to have higher calprotectin levels than healthy controls. The authors suggest that calprotectin may be a marker of disease severity rather than a marker for specific inflammatory subtypes [34]. Lee and colleagues found that S100A9 levels were higher in sputum from patients with severe uncontrolled asthma with neutrophilic inflammation than in sputum from eosinophilic and paucigranulocytic groups [37]. Together, these studies show that the role of S100A family proteins may vary according to the inflammatory context in the asthmatic airways. The role of S100A family is not clear in T2-high setting as it may favor or regulate T2 inflammation depending on the context [38–40]. Nevertheless, S100A molecules appear of cardinal importance in T17-high setting [33, 35, 37, 41]. Thus, BT-induced downregulation of S100A family proteins could imply modulation of more than one inflammatory pathway further supporting its ability to act on broader pathophysiological mechanisms rather than a specific endotype. Östling et al. reported an enrichment of canonical pathways associated to T17 biology (Role of IL-17A in psoriasis, role of IL-17F in allergic inflammatory airway diseases), TLR signaling, iNOS signaling and inflammasome in bronchial epithelial brushings of a subset of asthmatics. This was characterized by an upregulation of *S100A8* and *S100A9* amongst other genes [41]. Therefore, there are similarities in the gene signature identified by Ostling et al. in a subset of asthmatics and the gene signature we identified to be modulated by BT in our population. S100A7 is upregulated in response to IL-22, a Th17 cytokine in BECs [42]. S100A8 and S100A9 can promote mucus hypersecretion typical of asthma in BECs, which is consistent with our previously published works documenting reduced MUC5AC post-BT along with IL-13+ cells in bronchial biopsies collected from a similar cohort [21, 43]. Further, we showed that S100A7 and S100A8 expressions might be indicators of disease severity as measured by lower ACSS score and that patients with a greater S100A protein decrease had more of these proteins in their airway tissues prior to BT. This goes along with the lines of studies reporting higher S100A9 or calprotectin expression in serum with lower FEV_1_/FVC ratio, increased airway hyper-reactivity, or uncontrolled asthma [32, 34, 35]. Our results are consistent with current literature.

S100A family proteins are ligands of receptor for advanced glycation end products (RAGE) and of toll-like receptor 4 (TLR4). They are believed to promote inflammation through these pathways [30]. A house dust mite (HDM) sensitized RAGE-KO mouse model showed that airway structural cell expression of RAGE contributes to IL-33 accumulation and further ILC2 accumulation [44]. In other assays conducted in mice, calprotectin secretion was largely dependent upon RAGE signaling when stimulated with T2 cytokines (IL-4, 5 and 13) [45]. TLR4 signaling has also been shown to contribute to T2 inflammation. By selectively inactivating TLR4 on airway structural cells, a study group showed that airway epithelium of HDM stimulated mice produced more T2 alarmins (e.g. IL-25, IL-33, TSLP) and contributed to dendritic cells activation if they expressed TLR4 [46]. Exposition of normal BECs to *Alternaria* allergens induced TSLP and IL-25 secretion. Adding calprotectin further enhanced secretions of these cytokines [38]. A study using an asthma murine model challenged with *A. fumigatus* mixed with OVA showed that neutralizing antibodies against S100A8 and S100A9 alleviated airway inflammation and eosinophil recruitment [39].

On the other hand, some studies have reported beneficial effects for S100A8 and/or S100A9 expressions. For instance, a study using a calprotectin-deficient mouse model sensitized to *Alternaria alternata* showed that allergen exposition did increase S100A9 expression in the lung of wild type mice though the deficient mice displayed a worsened T2 inflammation and bronchial hyper-responsiveness [40]. Administering recombinant S100A8 to ovalbumin sensitized rats reduced pulmonary resistance and bronchial hyper-responsiveness. Further, in vitro administration of recombinant S100A8 on isolated tracheal rings from the same rat model reduced airway smooth muscle contractility [47].

CD36 is a scavenger receptor interacting with a broad range of ligands such as thrombospondin, collagen, long chain fatty acids and bacterial diacylated lipopeptides [48]. CD36 can associate with TLR2/6 or TLR4/6 complexes and favor immune response in context of infection or sterile inflammation [49, 50]. Further, calprotectin-arachidonate complex are recognized by CD36 receptor which could further favor arachidonate uptake and eicosanoids production by epithelial cells [51]. We observed a reduction of S100A family and of two of its receptors (TLR4 and CD36) in BECs post-BT; suggesting that BT benefits might be related to the reduced signaling pathways involving S100A family, TLR4 and CD36. Though, the role of S100A family proteins in TLR and CD36 signaling in the context of severe asthma remains unclear and needs further investigation.

We investigated the effect of BT on the epithelial expression of the trio of alarmins *IL-25* and *IL-33* and *TSLP* which are well documented in asthma as well as on *hBD2*, an antimicrobial peptide [52, 53]. Interestingly, we found a downregulation of *TSLP* and of *hBD2* in parallel to *S100A* family gene expressions. Though TSLP was classically associated to eosinophilic inflammation, it was more recently shown to be involved in non-eosinophilic inflammation and remodeling [54]. The results of recent clinical trials support this idea. Indeed, treatment with anti-TSLP reduced the exacerbation rate of patients with and without features of eosinophilic inflammation [25]. Considering that S100A8, S100A9, TSLP and hBD2 can be elicited by stimuli of various origins (allergens, pollutants, bacteria, cytokines), our results suggest that BT might act on mediators involved in broader pathophysiological mechanisms rather than targeting a specific endotype [38, 53, 54]. This is supported by other studies reporting better asthma control post-BT in patients with and without T2 inflammation [4, 5]. This might be a downstream effect of the BT-induced renewal of the epithelium and subsequent improved integrity [21, 22]. The later might improve the ability of the epithelium to orchestrate a proper response to external stimuli.

Severe asthma is associated with greater ASM mass with greater CXCL8 and eotaxin ASM expression [55, 56]. ASM can further stimulate the epithelium to produce various cytokines [57]. Many studies showed that BT induced long lasting ASM ablation [14–20]. It is not clear to which mechanism(s) S100A family downregulation may be attributed. It might be related to improvement in epithelial integrity leading to proper response to external stimuli and reduced production of alarmins and their receptors; ASM ablation reducing the need for contractility modulating proteins or ASM ablation leading to reduced production of chemokines and modulation of inflammation resulting in proper epithelial priming.

Taken together our results support the idea that the S100A family proteins and their related signaling pathways play a relevant role in severe asthma pathophysiology and that a reduced expression might be an indicator of response to BT treatment. They also support that BT, through its effects on airway structural cells, contributes to modulate local innate immune response and inflammation. The nature of BT treatment (heat delivery) and current evidence support that its beneficial effects may not be solely attributable to ASM ablation. For instance, we previously showed that BT induced the renewal of the ciliated cell layer, lastingly increased the number of basal progenitor cells and reduced the production of MUC5AC. This is accompanied by an increase in proliferating epithelial cells in the first two months [4, 21, 58] post-treatment while this goes back to basal level more than 1 year post treatment [21]. Further, in vitro experiments exposing epithelial cell cultures to heat as a proxy of BT showed that it may increase proliferation of epithelial cells and modulate their expression of differentiation markers [58, 59]. While in vitro heat treatment may be useful in assessing the effect of BT on epithelial cells, this only captures acute effect of heat which might not reflect long-lasting disease modifying mechanisms. We therefore believe that the current study assessing effect of BT on S100A family is more likely related to lasting clinical benefits.

Though the exact role of S100A family proteins in asthma pathophysiology is still not clear, higher expression is associated with disease. Further studies are needed to better understand the role of S100A family proteins and their receptors in relation to asthma pathophysiology which might be relevant in treatment monitoring.

Western Blots of selected S100A family receptors (RAGE, TLR4, CD36) on top with associated graphical representations below.

### Supplementary Information

**Additional file 1:** Supplementary method.

**Additional file 2: Table S3.** Down and up regulated genes in BECs post-BT.

**Additional file 3: Table S4.** Canonical pathways of genes modulated by BT.

**Additional file 4: Figure S1.** Purity validation of BEC. Shows a representative brightfield image of BEC culture (top) and a representative immunofluorescence for DAPI (blue) and pan-cytokeratin (green) (bottom).

**Additional file 5: Figure S2.** Transcriptomic analysis and canonical pathways of differentially expressed genes in BECs pre- and post-BT. **A**. Principal component analysis (PCA) 3-dimensional plots representing the transcript expression patterns of the different samples (pre- and post-BT: blue dots and red dots respectively). Each dot represents a sample showing two distinct transcript expression profiles. **B.** The main canonical pathways enriched in samples modulated by BT. **C.** Representation of some S100A genes in the IL-17 signaling pathway. Genes shown in green are down-regulated post-BT.

**Additional file 6: Figure S3.** Correlation analysis of S100A family proteins in tissues. **A.** Correlations of epithelial S100A family protein expressions with BT-induced expression changes. Y axis displays the expression differences post-BT (expression post-BT minus expression pre-BT). **B.** Correlations of epithelial S100A family proteins expression with ACSS score. ACSS is a local equivalent to ACQ.

**Additional file 7: Figure S4.** Gene expression of IL-25 and IL-33, TSLP and hBD2 in BECs of severe asthmatic patients pre- and post-BT. TSLP and hBD2 gene expressions decreased post-BT while no significant change was observed for IL-25 and IL-33.

## Data Availability

Transcriptomic data are accessible at the gene expression Omnibus (GEO) repository (https://www.ncbi.nlm.nih.gov/geo) with the provisional accession series number GSE216617.
