# Supplementary material for: S100A alarmins and thymic stromal lymphopoietin (TSLP) regulation in severe asthma following bronchial thermoplasty
Source: Respir Res. 2023 Nov 23;24:294. doi: 10.1186/s12931-023-02604-1 (PMC10668474; doi:10.1186/s12931-023-02604-1)
Supplement: Supplementary file 1 — Additional file 1: Supplementary method. [file 12931_2023_2604_MOESM1_ESM.docx]

**S100 A alarmins and Thymic stromal lymphopoietin (TSLP) regulation in severe asthma following bronchial thermoplasty**

Pierre-Alexandre Gagnon, Martin Klein, John De Vos, Sabrina Biardel, Andréanne Côté, Krystelle Godbout, Michel Laviolette, Catherine Laprise, Said Assou and Jamila Chakir.

**Online data supplement**

# Supplementary method

**Bronchial epithelial Cells culture**

Bronchial epithelial cells (BEC) isolated from bronchial biopsies of healthy controls (n=7) and severe asthmatics pre-BT (n=12) and 12-18 months post-BT (n=6) were cultivated in a combination of Dulbecco’s modified Eagle’s medium (DMEM) (Thermo Fisher Scientific, Massachusetts, USA) with Ham’s F12 (Thermo Fisher Scientific) in a 3:1 proportion supplemented with 10 ng/ml human epidermal growth factor (Austral biologicals, California, United-States), 24.3µg/ml adenine (Sigma Aldrich, Missouri, USA), 5µg/ml crystallized bovine insulin (Sigma Aldrich), 5µg/ml human transferrin (Sigma Aldrich), 2x10^-9^ M 3,3’5’ triiodo-L-thyronin (Sigma Aldrich), 0.4µg/ml hydrocortisone (VWR, Pennsylvania, USA), 8.47ng/ml cholera toxin (Sigma Aldrich), 10% fetal bovine serum (FBS) (VWR), 20 U/ml penicillin (Thermo Fisher Scientific), 20 ug/ml streptomycin (Thermo Fisher Scientific) and 25 ng/ml amphotericin B (Sigma Aldrich) (1). Cells were cultured until they reached over 90% confluence. They were then collected in the appropriate lysis buffer and stored at -80°C until needed.

**Microarray analysis, data processing and bioinformatics analysis**

RNA was isolated from 1×10^6^ airway epithelial cells isolated pre- and post-BT using RNeasy plus mini kit (Qiagen, California, USA). Total RNA was quantified using a NanoDrop Spectrophotometer ND-1000 (NanoDrop Technologies, Inc. Delaware, USA) and its integrity was assessed using a 2100 Bioanalyzer (Agilent Technologies, California, USA). Sense-strand cDNA was synthesized from 94 ng of total RNA, and fragmentation and labeling were performed to produce ss-cDNA with the GeneChip® WT Terminal Labeling Kit according to manufacturer’s instructions (Thermo Fisher Scientific). After fragmentation and labeling, 2.8 µg cDNA was targeted to be hybridized on Clariom™S HT, human (Thermo Fisher Scientific) and processed on a GeneTitan® instrument (Thermo Fisher Scientific) for Hyb-Wash-Scan automated workflow at the McGill University and Genome Quebec Innovation Centre (www.genomequebec.mcgill.ca). [RNA processing](https://www.sciencedirect.com/topics/biochemistry-genetics-and-molecular-biology/rna-processing) steps (RNA extraction, cDNA synthesis, cDNA fragmentation and labeling, and chip hybridization) were performed in parallel (pre- and post-BT) for each pair of epithelial cell samples to minimize technical variability. The raw image files (CEL format) generated from the analysis of the scanned image were used for the statistical analysis.

After image processing, the CEL files were transferred to transcriptome analysis console (TAC) version 4.0 (Affymetrix) to analyze the expression pattern of the transcripts using the robust multi-array average (RMA) algorithm and to construct a principal component analysis (PCA), the volcano plot and the heat-map. Different transcript levels with a significant *p*-value <0.05 and fold changes of 2 or higher and of -2 or lower were retained. The probe sets were annotated using the Affymetrix annotation file from Netaffx (<http://www.netaffx.com>). The gene ontology (GO) enrichment analysis, the pathways, and networks of differentially expressed transcripts between pre- and post-BT samples were analyzed using Ingenuity Pathway Analysis (IPA) software (IPA; Qiagen Inc., <http://www.ingenuity.com>). All our data are accessible at the gene expression Omnibus (GEO) repository (<https://www.ncbi.nlm.nih.gov/geo>) with the provisional accession series number GSE216617.

**Real-Time Quantitative PCR Analysis**

Total RNA extraction was performed using illustra RNAspin Mini RNA Isolation Kit (Sigma-Aldrich) according to manufacturer’s instructions. Total RNA quantity and purity were measured using nanodrop 2000 spectrophotometer (Thermo Fisher Scientific). One μg of total RNA was reverse transcribed using High-Capacity cDNA Reverse Transcription Kit (Thermo Fisher Scientific). qPCRs were performed in triplicate using iQ SYBR green Supermix (Biorad, California, USA). qRT-PCR were performed using C1000 thermal cycler with CFX-96 qPCR system (BioRad). Relative gene expression was normalized on GAPDH of healthy individuals’ expression using ΔΔCt method (2). Supplementary Table E1 gives further information about targeted genes and primer pairs.

**Protein extraction and Western blot**

Cells were lysed in cell lysis buffer (New England Biolabs, Massachusetts, USA) to which complete, mini EDTA free protease inhibitor cocktail (Sigma Aldrich), protease inhibitor (Sigma Aldrich) and phosphatase inhibitor (Sigma Aldrich) were added. Total protein concentration was measured using a Bradford assay. Twenty to 50ug of total proteins were loaded on 12,5-20% acrylamide gel for SDS-PAGE. Proteins were transferred on Polyvinylidene difluoride (PVDF) membranes which were then blocked with 5% non-fat milk or 1% BSA for 1h at room temperature. Membranes were incubated with primary antibody overnight at 4°C. Incubation with secondary antibody was done for 1h at room temperature. PVDF membranes were washed using Tris Buffered Saline (TBS)-Tween 0,05% after each incubation with an antibody. Revelation was done using Immobilion Western chemiluminescent HRP substrate (Sigma Aldrich) and Chemidoc universal hood III (Biorad). Afterwards, protein expression was measured by densitometry using Image Lab (Biorad) and normalized on B-actin expression. Supplementary Table E2 gives further details about antibodies used in the experiments.

**Immunohistochemistry (IHC)**

Five µm sections of paraffin embedded bronchial biopsies (n=20) were stained using EXPOSE mouse and rabbit specific HRP/AEC detection IHC kit (Abcam, Cambridge, United-Kingdom) according to manufacturer’s instructions. Briefly, following deparaffinization, antigen retrieval was performed by microwaving slides in citrate buffer pH 6 for 13 minutes. Sections were counterstained using hematoxylin (Sigma-Aldrich). Slides were scanned using Axioscan 7 (Zeiss, Oberkochen, Germany). Positively stained mucosal area was measured as a percentage of total non-metaplastic mucosal area using ImageJ (NIH, V1.53f51) as we previously reported (3). One to 5 biopsies were used for each patient. Protein expression is reported as mean values of each follow-up session. Supplementary Table E2 gives further details about antibodies used in the experiments.

**Table S1: primers used for quantitative RT-PCR.**

| Gene | NCBI accession number | Primers sequences | Annealing temperature (°C) | Fragment size (bp) |
| --- | --- | --- | --- | --- |
| *GAPDH (4)* | NM_001357943 | Forward: GGTATCGTCGAAGGACTCATGAC  Reverse: ATGCCAGTGAGCTTCCCGTTCAGC | 60 | 188 |
| *S100A7* | NM_002963 | Forward: CACAAATTACCTCGCCGAT  Reverse: GGTAGTCTGTGGCTATGTCTC | 60 | 107 |
| *S100A8* | NM_002964 | Forward: CGTCTGGTTCAAAGAGTTGGA  Reverse: GCCACGCCCATCTTTATCAC | 60 | 89 |
| *S100A9* | NM_002965 | Forward: AAGAGCTGGTGCGAAAAGAT  Reverse: TCTGCATTTGTGTCCAGGTC | 60 | 100 |
| *IL25* | NM_022789 | Forward: AGCCGGTTCAAGTCTCTGTC  Reverse: CAGAGTCCTGTAGGGCCAGT | 55 | 91 |
| *IL33* | NM_001199640 | Forward: TTATCATAAGGCCAGAGCGG  Reverse: GAACACAGCAAGCAAAGCCT | 60 | 107 |
| *sfTSLP* | NM_138551 | Forward: CGTAAACTTTGCCGCCTATGA  Reverse: TTCTTCATTGCCTGAGTAGCATTTAT | 62 | 186 |
| *hBD2* | NM_004942 | Forward: TGTGGTCTCCCTGGAACAAAAT  Reverse: GTCGCACGTCTCTGATGAGG | 60 | 105 |

**Table S2: Antibodies used for Western blot and immunohistochemistry.**

| **Target** | **Clone** | **Host** | **Dilution (application)** | **manufacturer** |
| --- | --- | --- | --- | --- |
| S100A7 | 47C1068 | Mouse | 1:500 (WB); 1:50 (IHC) | Novus Biologicals |
| S100A8 | Polyclonal | Rabbit | 1:1000 (WB); 1:100 (IHC) | Novus Biologicals |
| S100A9 | EPR3555 | Rabbit | 1:1000 (WB); 1:400 (IHC) | Abcam |
| RAGE | D1A12 | Rabbit | 1:1000 (WB) | Cell Signaling Technologies |
| TLR4 | Polyclonal | Rabbit | 1:1000 (WB) | Santa Cruz Biotechnology |
| CD36 | Polyclonal | Rabbit | 1:1000 (WB) | Novus Biologicals |
| B-actin | AC-15 | Mouse | 1:5000 (WB) | Millipore Sigma |
| Mouse Ig-HRP | polyclonal | Goat | 1:2 of primary antibody concentration (WB) | BD Bioscience |
| Rabbit Ig-HRP | polyclonal | Goat | 1:2 of primary antibody concentration (WB) | Millipore Sigma |

# Supplementary results

**Table S3. Down and up regulated genes in BECs post-BT.**

**Table S4. Canonical pathways of genes modulated by BT.**

**Figure S1. Purity validation of BEC.** Shows a representative brightfield image of BEC culture (top) and a representative immunofluorescence for DAPI (blue) and pan-cytokeratin (green) (bottom)

**Figure S2. Transcriptomic analysis and canonical pathways of differentially expressed genes in BECs pre- and post-BT. A**. Principal component analysis (PCA) 3-dimensional plots representing the transcript expression patterns of the different samples (pre- and post-BT: blue dots and red dots respectively). Each dot represents a sample showing two distinct transcript expression profiles. **B.** The main canonical pathways enriched in samples modulated by BT. **C.** Representation of some S100A genes in the IL-17 signaling pathway. Genes shown in green are down-regulated post-BT.

**Figure S3.** **Correlation analysis of S100A family proteins in tissues.** **A.** Correlations of epithelial S100A family protein expressions with BT-induced expression changes. Y axis displays the expression differences post-BT (expression post-BT minus expression pre-BT). **B.** Correlations of epithelial S100A family proteins expression with ACSS score. ACSS is a local equivalent to ACQ

**Figure S4. Gene expression of IL-25 and IL-33, TSLP and hBD2 in BECs of severe asthmatic patients pre- and post-BT.** TSLP and hBD2 gene expressions decreased post-BT while no significant change was observed for IL-25 and IL-33.

# Supplementary References

1. Semlali A, Jacques E, Plante S, Biardel S, Milot J, Laviolette M, Boulet LP, Chakir J. TGF-beta suppresses EGF-induced MAPK signaling and proliferation in asthmatic epithelial cells. *Am J Respir Cell Mol Biol* 2008; 38: 202-208.

2. Livak KJ, Schmittgen TD. Analysis of relative gene expression data using real-time quantitative PCR and the 2(-Delta Delta C(T)) Method. *Methods* 2001; 25: 402-408.

3. Haj Salem I, Gras D, Joubert P, Boulet LP, Lampron N, Martel S, Godbout K, Chanez P, Laviolette M, Chakir J. Persistent Reduction of Mucin Production after Bronchial Thermoplasty in Severe Asthma. *Am J Respir Crit Care Med* 2019; 199: 536-538.

4. Semlali A, Witoled C, Alanazi M, Rouabhia M. Whole cigarette smoke increased the expression of TLRs, HBDs, and proinflammory cytokines by human gingival epithelial cells through different signaling pathways. *PLoS One* 2012; 7: e52614.

5. Jiang M, Wu N, Xu B, Chu Y, Li X, Su S, Chen D, Li W, Shi Y, Gao X, Zhang H, Zhang Z, Du W, Nie Y, Liang J, Fan D. Fatty acid-induced CD36 expression via O-GlcNAcylation drives gastric cancer metastasis. *Theranostics* 2019; 9: 5359-5373.
