# Supplementary figures and images for: S100A alarmins and thymic stromal lymphopoietin (TSLP) regulation in severe asthma following bronchial thermoplasty
Source: Respir Res. 2023 Nov 23;24:294. doi: 10.1186/s12931-023-02604-1 (PMC10668474; doi:10.1186/s12931-023-02604-1)

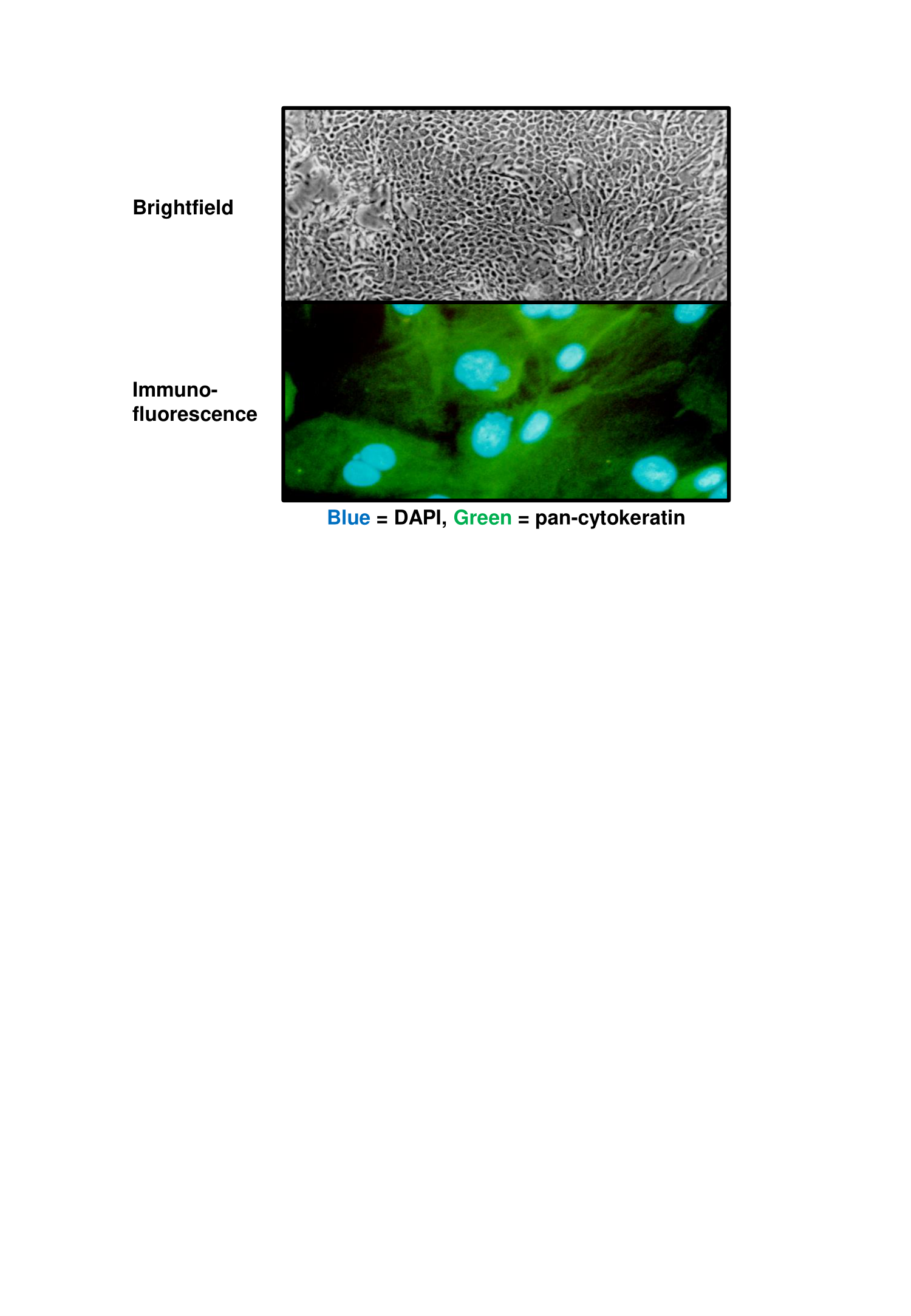

Supplement: Supplementary file 4 — Additional file 4: Figure S1. Purity validation of BEC. Shows a representative brightfield image of BEC culture (top) and a representative immunofluorescence for DAPI (blue) and pan-cytokeratin (green) (bottom). [file 12931_2023_2604_MOESM4_ESM.tiff]

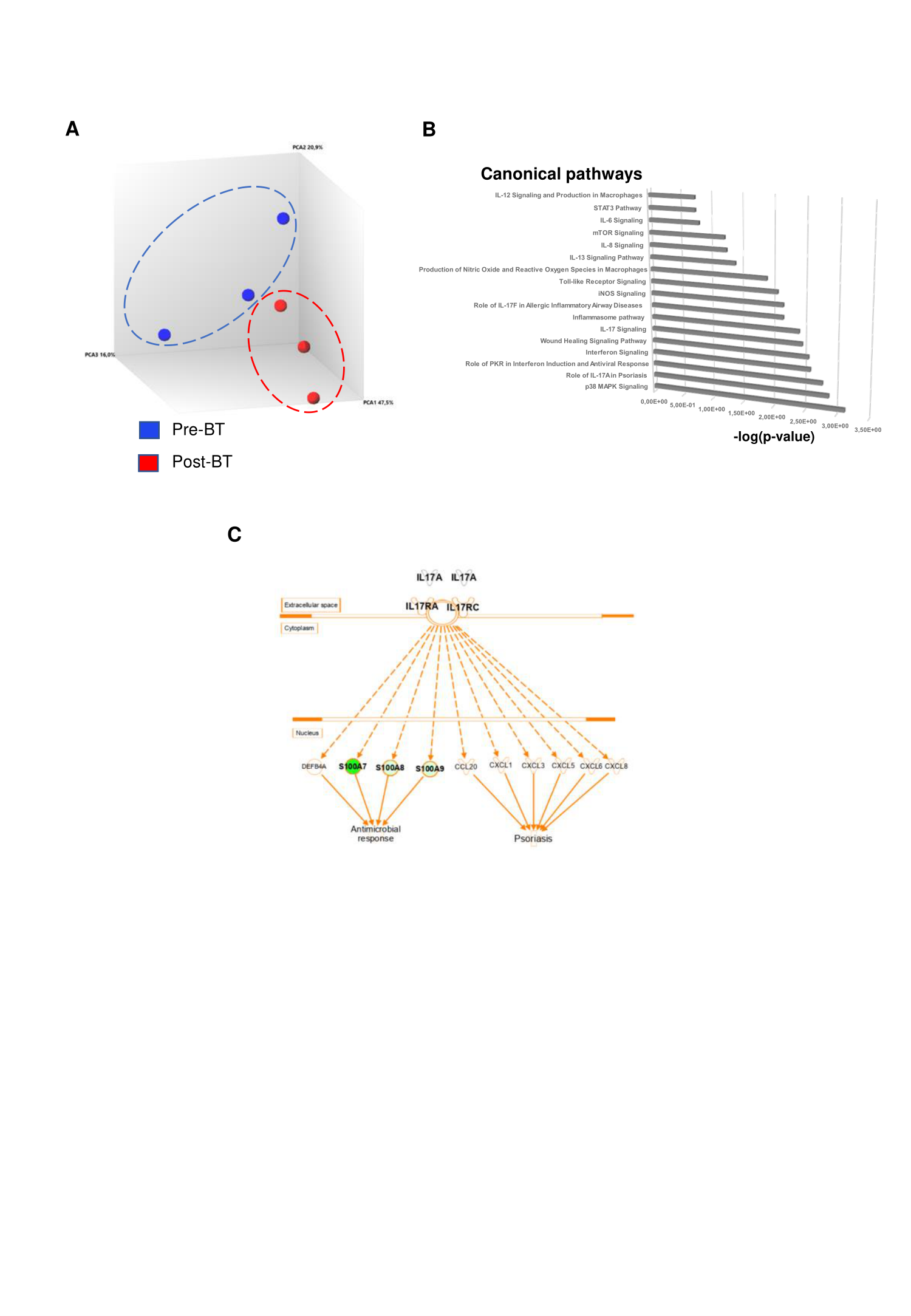

Supplement: Supplementary file 5 — Additional file 5: Figure S2. Transcriptomic analysis and canonical pathways of differentially expressed genes in BECs pre- and post-BT. A. Principal component analysis (PCA) 3-dimensional plots representing the transcript expression patterns of the different samples (pre- and post-BT: blue dots and red dots respectively). Each dot represents a sample showing two distinct transcript expression profiles. B. The main canonical pathways enriched in samples modulated by BT. C. Representation of some S100A genes in the IL-17 signaling pathway. Genes shown in green are down-regulated post-BT. [file 12931_2023_2604_MOESM5_ESM.tiff]

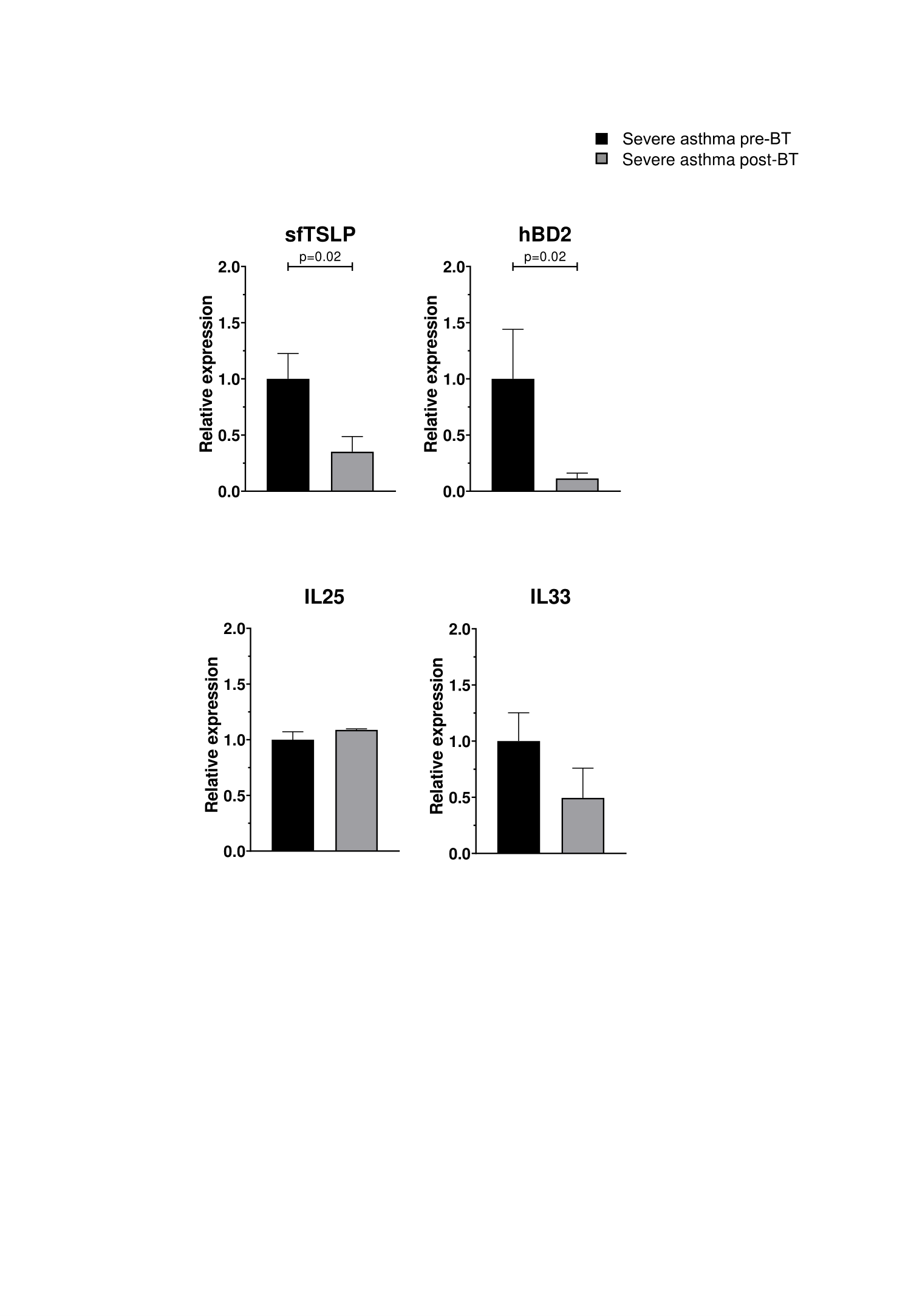

Supplement: Supplementary file 7 — Additional file 7: Figure S4. Gene expression of IL-25 and IL-33, TSLP and hBD2 in BECs of severe asthmatic patients pre- and post-BT. TSLP and hBD2 gene expressions decreased post-BT while no significant change was observed for IL-25 and IL-33. [file 12931_2023_2604_MOESM7_ESM.tiff]
